# Spectral CT-based radiomics signature for distinguishing malignant pulmonary nodules from benign

**DOI:** 10.1186/s12885-023-10572-4

**Published:** 2023-01-26

**Authors:** Hang Xu, Na Zhu, Yong Yue, Yan Guo, Qingyun Wen, Lu Gao, Yang Hou, Jin Shang

**Affiliations:** 1 Department of Radiology, Shengjing Hospital of China Medical University, Shenyang, 110004 China; 2 Department of Radiation Oncology, Nanfang Hospital of Southern Medical University, Guangzhou, 510000 China; 3 GE Healthcare, Shenyang, 110004 China; 4 Department of Radiology, Jining First People’s Hospital, Jining, 272000 China; 5 Department of Radiology, Liaoning Province Cancer Hospital, Shenyang, 110801 China

**Keywords:** Solitary pulmonary solid nodules, Discrimination, Dual-layer spectral detector CT, Radiomics

## Abstract

**Objectives:**

To evaluate the discriminatory capability of spectral CT-based radiomics to distinguish benign from malignant solitary pulmonary solid nodules (SPSNs).

**Materials and methods:**

A retrospective study was performed including 242 patients with SPSNs who underwent contrast-enhanced dual-layer Spectral Detector CT (SDCT) examination within one month before surgery in our hospital, which were randomly divided into training and testing datasets with a ratio of 7:3. Regions of interest (ROIs) based on 40-65 keV images of arterial phase (AP), venous phases (VP), and 120kVp of SDCT were delineated, and radiomics features were extracted. Then the optimal radiomics-based score in identifying SPSNs was calculated and selected for building radiomics-based model. The conventional model was developed based on significant clinical characteristics and spectral quantitative parameters, subsequently, the integrated model combining radiomics-based model and conventional model was established. The performance of three models was evaluated with discrimination, calibration, and clinical application.

**Results:**

The 65 keV radiomics-based scores of AP and VP had the optimal performance in distinguishing benign from malignant SPSNs (AUC_65keV-AP_ = 0.92, AUC_65keV-VP_ = 0.88). The diagnostic efficiency of radiomics-based model (AUC = 0.96) based on 65 keV images of AP and VP outperformed conventional model (AUC = 0.86) in the identification of SPSNs, and that of integrated model (AUC = 0.97) was slightly further improved. Evaluation of three models showed the potential for generalizability.

**Conclusions:**

Among the 40-65 keV radiomics-based scores based on SDCT, 65 keV radiomics-based score had the optimal performance in distinguishing benign from malignant SPSNs. The integrated model combining radiomics-based model based on 65 keV images of AP and VP with Z_eff-AP_ was significantly superior to conventional model in the discrimination of SPSNs.

**Supplementary Information:**

The online version contains supplementary material available at 10.1186/s12885-023-10572-4.

## Introduction

At present, radiomics research based on traditional CT has revealed the potential to differentiate benign and malignant pulmonary nodules [1–9]. So far, few radiomics-based studies have applied different Dual-energy-CT (DECT) images for characterizing tumors, where the rich and additional quantitative information on the energy-dependent attenuation changes in different tissues could potentially improve performance of predictive models [10–12]. As an emerging and exciting DECT, Dual-layer Spectral Detector CT (SDCT) is proved to be a promising technology in oncologic identification [13–16]. Firstly, it collects high and low energy information and acquires in-phase, temporally synchronized, and homologous photons in a conventional CT scanning, which improves the accuracy of data collection. Secondly, SDCT exploits anti-correlated noise suppression [17], leading to a constantly low noise level [18]. Thirdly, SDCT eliminates the requirement to pre-select patients or change clinical workflow [19], permitting evaluation of incidentally discovered findings. Recent studies have shown that spectral quantitative parameters could further improve the discriminative ability of pulmonary nodules [20–24], such as CT values of 40 keV monochromatic images (CT_40 keV_), the slope of the spectral Hounsfield Unit curve (λ_HU_), iodine concentration (IC), normalized iodine concentration (NIC), and the differences in NIC between the proximal and the distal regions in pulmonary nodules (dNIC). IC reflects the difference of blood supply within the lesions [22, 25, 26], and λ_HU_ presents the attenuation characteristics of different tissues [27]. Although the role of both in identifying benign and malignant nodules has been widely mentioned, studies on the selection of the optimal sequence of virtual monochromatic images (VMI) remain rare. Hence, we aimed to select the optimal sequence of VMI based on the latest SDCT for the first time and develop a spectral CT radiomics-based signature to differentiate solitary pulmonary solid nodules (SPSNs).

## Materials and methods

### Patients

**Fig. 1:**
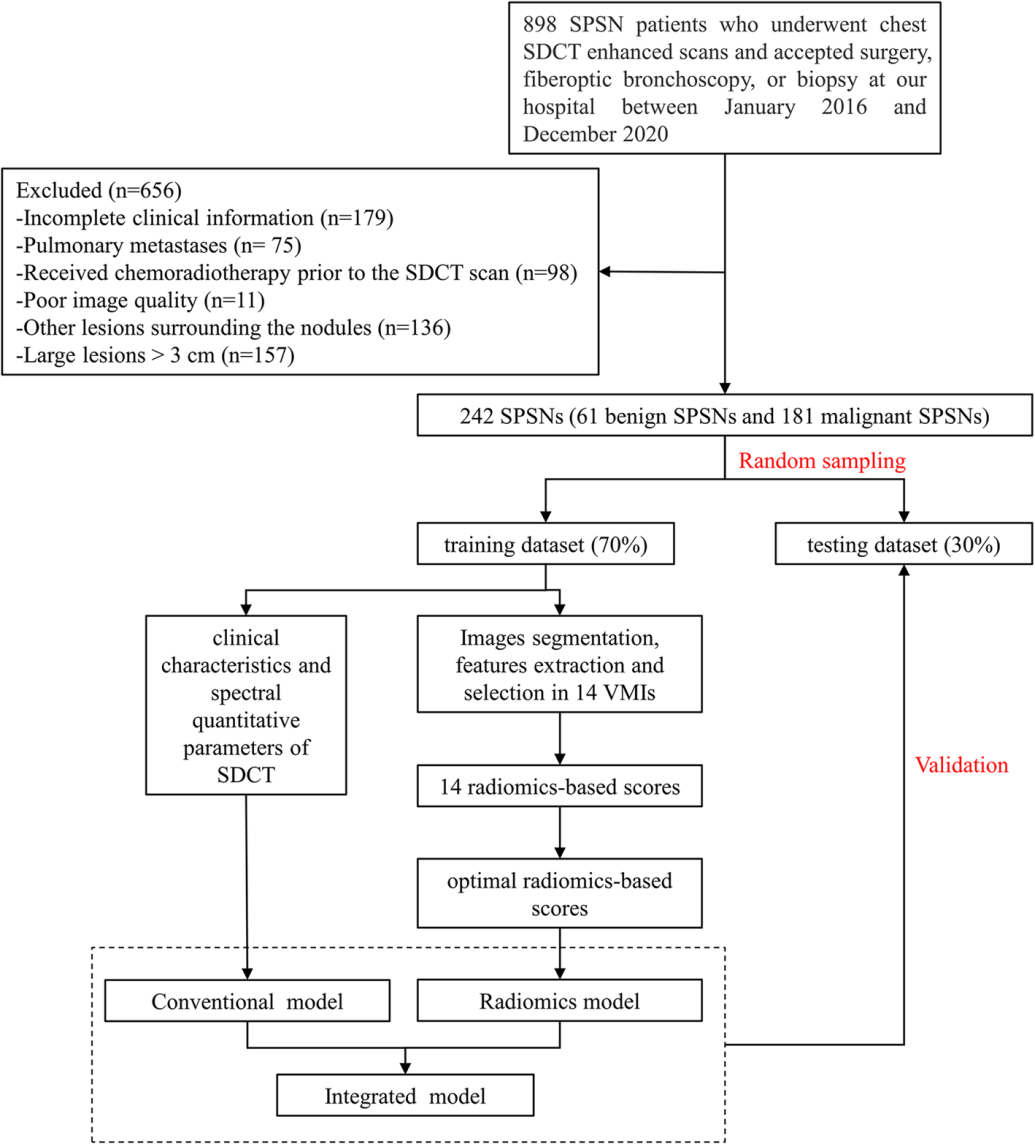
Overview workflow of this study. SDCT, dual-layer Spectral Detector CT; SPSN, solitary pulmonary solid nodule; VMI, virtual monochromatic images

The study population was retrospectively enrolled SPSNs patients who underwent contrast-enhanced SDCT examination within one month before surgery from our hospital between January 2016 and December 2020. Ultimately, 242 patients (average age 59.90 ± 10.55 years) were included. We collected 6 clinical risk factors including age, sex, smoking, carcinoembryonic antigen (CEA), cytokeratin 19 fragment 21–1 (CYFRA21-1), and neuron-specific enolase (NSE). The final cohort was randomly divided into training (*n* = 168) and testing datasets (*n* = 74) with a ratio of 7:3. An overview workflow of this study was shown in Fig. 1.

### Image acquisition

Contrast-enhanced chest scans were performed in both arterial phase (AP) and venous phase (VP) on dual-layer spectral detector CT (IQon Spectral CT, Philips Healthcare, Best, The Netherlands). The range of scan was from the thoracic inlet to the bottom of the thoracic cavity in order to cover all lung tissues. After a native chest scan, contrast agent (Iodixanol, 350 mg/mL, GE Healthcare, Ireland) was injected via the cubital vein with a power injector (Ulrich REF XD 2051), at a volume of 80 ml and flow rate of 2.5 mL/s. AP and VP scans were acquired at 25 and 60 s after the contrast agent injection. Spectral CT scan parameters were as follows: tube voltage = 120 kV; automatic tube current exposure control [Dose Right Index (DRI)] = 22; tube rotation time = 0.5 s; detector collimation = 64 × 0.625 mm; a reconstructed slice thickness = 0.9 mm; slice increment = 0.45 mm; field of view = 250 × 250 mm; image reconstruction matrix = 512 × 512. All original images were reconstructed as Spectral Base Image (SBI) datasets with reconstructed slice thickness of 1 mm and increment of 1 mm, then were transmitted to a dedicated post-processing workstation of spectral CT (IntelliSpace Portal 6.5, Philips Healthcare, Best, The Netherlands) for image analysis.

### Spectral quantitative parameter measurement

Quantitative parameters of spectral CT were measured on the best-displayed plane and the relative homogeneous area of lesions in 40-65 keV images of AP, VP, and conventional 120kVp in the dedicated post-processing workstation (IntelliSpace Portal 6.5, PhilipsHealthcare, Best, The Netherlands). The following quantitative measurements were performed twice, and the average value was calculated. Quantitative parameters were as follows: (I) IC of the lesion (IC_lesion_) and IC in the same layer of aorta (IC_aorta_), calculated NIC = IC_lesion_/IC_aorta_; (II) effective atomic number (Z_eff_); (III) CT values of 40 keV (CT_40keV_) and 80 keV monochromatic images (CT_80keV_); (IV) λ_HU_ =|CT_40keV_ − CT_80keV_|/(80–40).

### Images segmentation

The original CT images were imported into the image preprocessing module of Artificial Intelligence Kit (A.K., GE Healthcare, China), then which were preprocessed to ensure that the voxel points of the images were isotropic. The preprocessed images were uploaded to ITK-SNAP software (http://www.itksnap.org), and the two-dimensional region of interest (ROI) was manually delineated on the single representative section that had the largest nodule area on CT images of 40 keV by a radiologist (with 14 years of experience in chest imaging diagnosis) who was blinded to the clinical data and histopathologic results. The copy-and-paste function was used to ensure that the position and size of the ROIs were consistent between different VMIs of SDCT.

### Feature extraction, selection, and screen of the optimal sequence of VMI

**Fig. 2:**
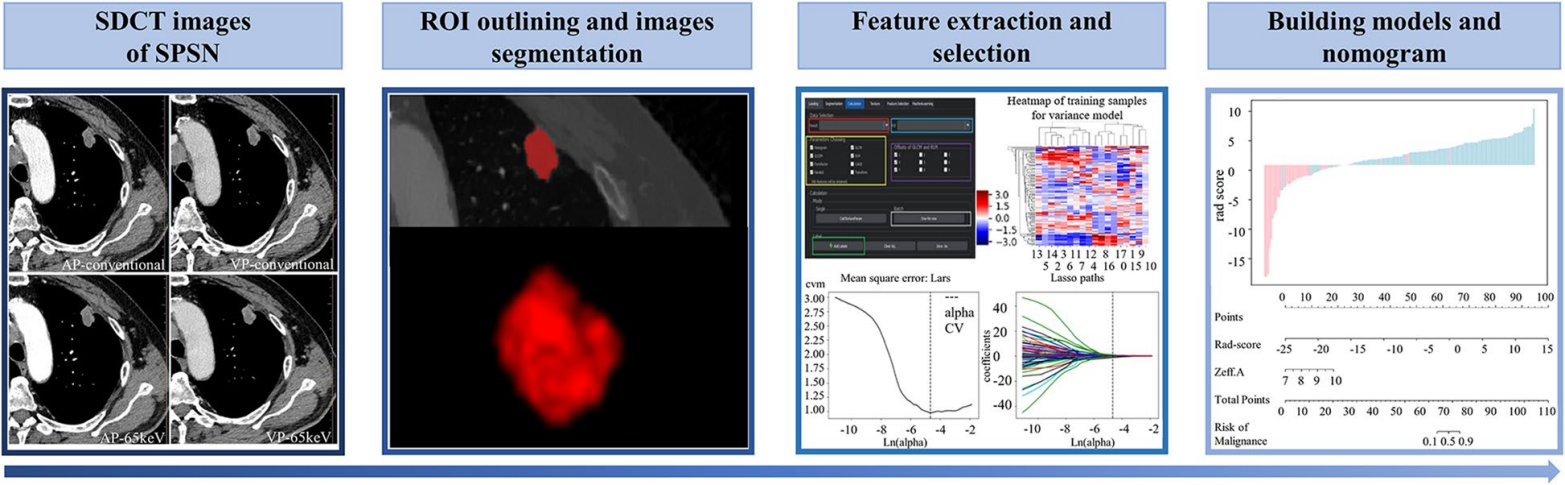
A flow chart displaying the process of building radiomics-based model in this study. AP, arterial phase; VP, venous phase; SDCT, dual-layer Spectral Detector CT; SPSN, solitary pulmonary solid nodule; ROI, region of interest

Radiomics features were extracted from ROIs with the reference of the image biomarker standardization initiative (IBSI) [28] using an artificial intelligence kit (A.K., GE Healthcare), and the data was standardized. In order to ensure stability and robustness of the radiomics features, 30 cases were randomly selected in a blinded manner. The same image segmentation process and feature extraction were conducted among the 30 cases by another radiologist (with 7 years of experience in chest imaging diagnosis). The intraclass correlation coefficient (ICC) was calculated to test the interobserver reproducibility of the radiomics features. Features with ICC value > 0.75 were considered a good agreement and were used for subsequent analysis [29]. The least absolute shrinkage and selection operator (LASSO) regression was conducted to select the optimal radiomics features with non-zero coefficients via ten-fold cross-validation. Finally, a total of 14 radiomics-based scores were respectively calculated based on optimal radiomics features of 40-65 keV images of AP, VP, and conventional 120kVp by multivariate logistic regression, then the optimal radiomics-based score was screened for subsequent modeling. A radiomics workflow was shown in Fig. 2.

### Models building

Significant clinical features and spectral CT quantitative parameters which were selected by univariate logistic regression and Spearman correlations analysis [30], were used to construct the clinical features and spectral CT quantitative parameters-based model. Then we defined it as conventional model. The above-mentioned optimal radiomics-based score was used to establish a radiomics-based model through multivariate logistic regression. Finally, an integrated model incorporating radiomics-based model with conventional model was built by univariate and multivariate logistic regression with stepwise selection method.

### Statistical analysis

All statistical analyses were performed with R software (version 3.5.1; http://www.Rproject.org). Quantitative data with normal distribution was presented as mean S ± SD; quantitative data with abnormal distribution was presented as median (25th, 75th percentile). Categorical variables were compared by chi-square test or the Fisher exact test; either Student’s t-test or Mann-Whitney U test was used for the continuous variables as appropriate. The level of significance was *p* ＜ 0.05.

Receiver-operating characteristic (ROC) curves were applied to assess the predictive ability of the models in distinguishing benign from malignant SPSNs, and accuracy, sensitivity, specificity, and the area under the curve (AUC) were calculated respectively. The DeLong test was used to compare the AUC between different models or different datasets. Calibration curves were plotted and the Hosmer-Lemeshow test was used to assess the fitness of the models. Decision curves were used to compare the clinical usefulness of models.

## Results

### Patient characteristics

A total of 242 patients’ clinical characteristics and spectral quantitative parameters from SDCT in the training and testing datasets were detailed in Table 1. There were 61 benign SPSNs and 181 malignant SPSNs in this cohort (Supplementary Materials).

**Table 1:** Clinical risk factors of the study population and quantitative parameters from SDCT in the training and testing datasets

Variables	**Training Dataset (*N* = 168)**			**Testing Dataset (*N* = 74)**			***p* value**
	Benign SPSNs (*N* = 42)	Malignant SPSNs (*N* = 126)	*p* value	Benign SPSNs (*N* = 19)	Malignant SPSNs (*N* = 55)	*p* value	
Male	15(35.71%)	70(55.56%)	0.026*	11(57.89%)	27(49.09%)	0.508	0.914
Age	58.00(49.95, 63.00)	62.00(54.95, 67.00)	0.035*	54.26 ± 11.32	60.87 ± 9.11	0.013*	0.559
Smoking	12(28.57%)	60(47.62%)	0.031*	8(42.11%)	25(45.45%)	0.800	0.802
CEA	2.40(1.40, 3.08)	4.36(2.20, 24.25)	< 0.001*	1.99(1.57, 3.06)	4.36(2.53, 25.51)	< 0.001*	0.836
CYFRA21-1	2.48(2.10, 2.88)	3.42(2.45, 6.16)	< 0.001*	2.20(1.71, 3.62)	3.99(2.31, 5.83)	0.001*	0.975
NSE	14.20(12.31, 15.81)	14.96(13.10, 19.00)	0.018*	13.76(12.48, 14.34)	14.90(12.87, 17.54)	0.089	0.399
IC_AP_	0.86(0.42, 1.57)	1.23(0.98, 1.66)	0.004*	0.84(0.64, 1.31)	1.36(1.17, 1.90)	< 0.001*	0.254
IC_VP_	1.23 ± 0.89	1.60 ± 0.52	0.014*	0.99 ± 0.68	1.82 ± 0.68	< 0.001*	0.302
NIC_AP_	0.10(0.04, 0.17)	0.13(0.10, 0.16)	0.010*	0.10(0.05, 0.13)	0.15(0.11, 0.19)	< 0.001*	0.293
NIC_VP_	0.27 ± 0.17	0.36 ± 0.10	0.003*	0.20 ± 0.12	0.41 ± 0.15	< 0.001*	0.567
CT_40keV-AP_	107.00(68.95, 152.96)	142.30(119.78, 181.00)	0.001*	105.40(78.08, 145.90)	154.10(130.84, 196.44)	< 0.001*	0.304
CT_40keV-VP_	137.94 ± 77.43	172.64 ± 44.60	0.008*	114.26 ± 61.03	187.63 ± 49.54	< 0.001*	0.551
λ_HU-AP_	1.45(0.70, 2.39)	2.08(1.64, 2.76)	0.003*	1.43(1.09, 2.19)	2.28(1.96, 3.17)	< 0.001*	0.247
λ_HU-VP_	2.05 ± 1.51	2.62 ± 0.88	0.026*	1.63 ± 1.14	3.03 ± 1.14	< 0.001*	0.235
Z_eff-AP_	7.87(7.60, 8.18)	8.07(7.94, 8.27)	0.002*	7.81 ± 0.33	8.22 ± 0.36	< 0.001*	0.164
Z_eff-VP_	7.92(7.58, 8.33)	8.18(8.06, 8.38)	0.001*	7.81(7.68, 8.20)	8.32(8.14, 8.48)	< 0.001*	0.239

*SDCT* Dual-layer spectral detector CT, *CEA* Carcinoembryonic antigen, *CYFRA21-1* Cytokeratin 19 fragment 21-1, *NSE* Neuron-specific enolase, *IC* Iodine concentration, *NIC* Normalized iodine concentration, *CT*_*40keV*_ CT values of 40 keV monochromatic images, *λ*_*HU*_ Dual-energy curve slope value, *Z*_*eff*_ Effective atomic number, *AP* Arterial phase, *VP* Venous phase

*p* values reflected the differences between benign SPSNs and malignant SPSNs, *p* values reflected the differences between the training and testing datasets, and *p* values and *p* values were computed by using Student’s t-test or Mann-Whitney U test for continuous variables and chi-square test or the Fisher exact test for categorical data

^*^ in the upper right indicates *p* < 0.05

### Feature selection and optimal VMI sequence screening

A total of 107 radiomics features with interobserver ICC value > 0.75 were extracted from ROIs based on 40-65 keV images of AP, VP, and conventional 120kVp respectively. Then the optimal radiomics features of each sequence remained respectively after LASSO, which were devoted to calculating 14 radiomics-based scores by multivariate logistic regression.

The diagnostic performance of 14 radiomics-based scores was detailed in Supplementary Table A2. Among these, the diagnostic performance based on 65 keV images in both AP and VP was the best in differentiating benign and malignant SPSNs in the training (AUC_65keV-AP_ = 0.94, AUC_65keV-VP_ = 0.92) and testing datasets (AUC_65keV-AP_ = 0.92, AUC_65keV-VP_ = 0.88), respectively. The optimal radiomics features of 65 keV radiomics-based scores in AP and VP were displayed respectively in Supplementary Table A3.

### Models building

**Fig. 3:**
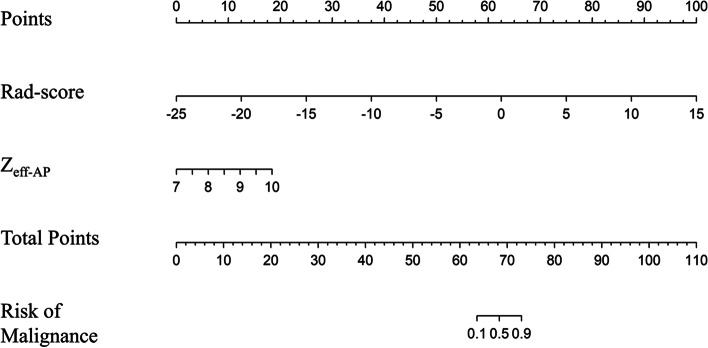
Developed integrated model nomogram. The integrated model nomogram was built in training dataset with Z_eff-AP_ and a Rad-score of the selected radiomics features incorporated. Z_eff-AP_, effective atomic number in arterial phase; Rad-score, the score of spectral CT-based radiomics model combining radiomics scores based on 65 keV images of arterial phase and venous phase

Among the 16 clinical characteristics and spectral quantitative parameters, 14 features were selected by univariate logistic regression, eight features (CEA, Z_eff-AP_, age, CYFRA21-1, NSE, CT_40keV-VP_, NIC_AP_, NIC_VP_) were remained after redundancy with Spearman correlation analysis, which were used to establish conventional model. The radiomics-based scores based on 65 keV images of AP and VP were combined to build the radiomics model by multivariate logistic regression. Combined conventional model with radiomics model, eight features (CEA, age, CYFRA21-1, CT_40keV-VP_, score of radiomics model_65keV_, Z_eff-AP_, NIC_VP_, NSE) were selected by univariate logistic regression, two features (score of radiomics model_65keV_, Z_eff-AP_) were remained after multivariate logistic regression, which were committed to developing an integrated model. We presented it as a nomogram in Fig. 3. The calculation formulas for three models were shown in Supplementary Materials.

### Performance evaluation

**Fig. 4:**
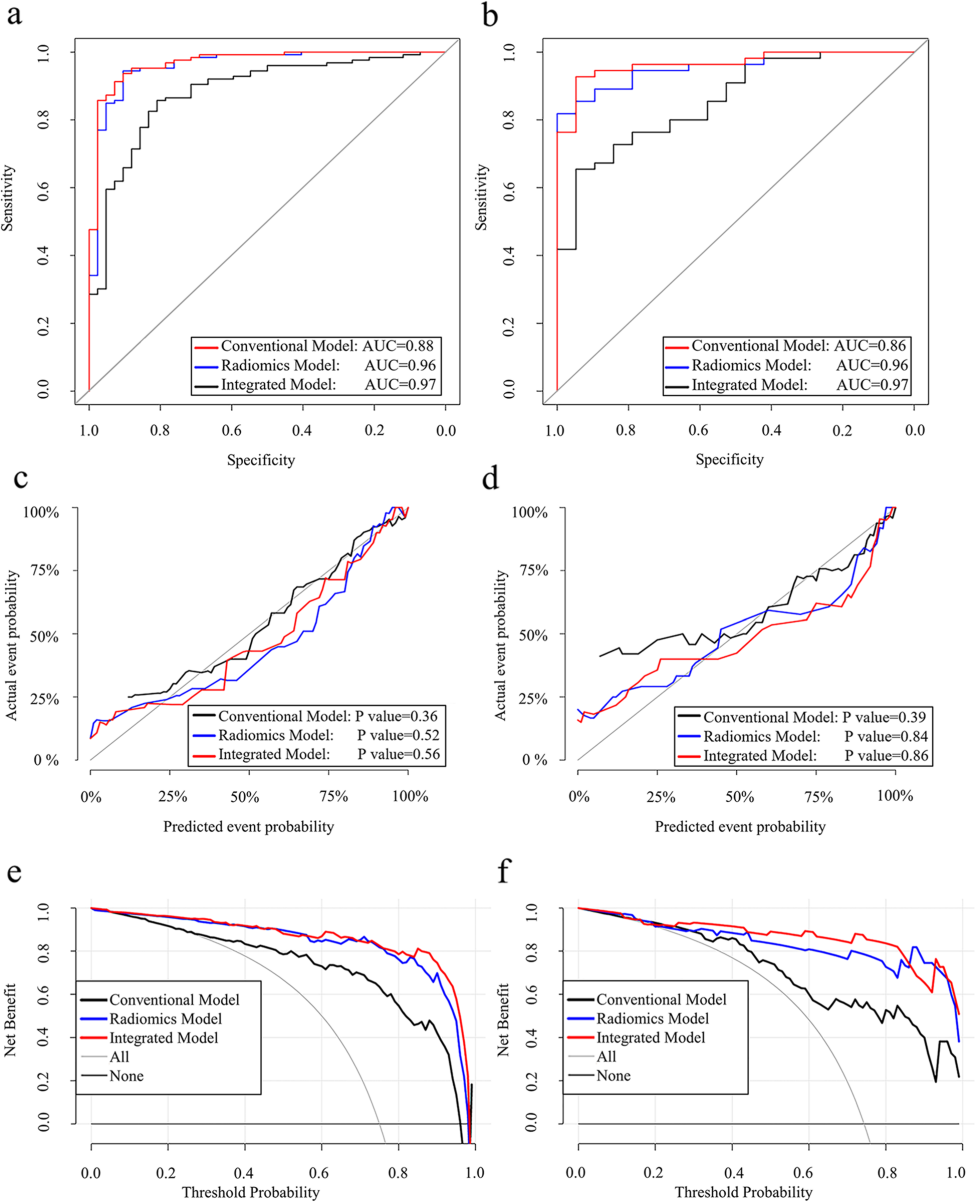
The performance of three models in distinguishing benign from malignant SPSNs. ROC curves for radiomics model, conventional model, and integrated model in training (**a**) and testing datasets (**b**). Calibration curves for radiomics model, conventional model, and integrated model in training (**c**) and testing datasets (**d**). The calibration curves described a good fitness of three models between prediction and observation of the benign and malignant SPSNs. The gray line represented the perfect prediction. A closer fitness to the gray line represented a well-calibrated model. Decision curves for radiomics model, conventional model, and integrated model in training (**e**) and testing datasets (**f**). Integrated model and radiomics model had higher net benefit than conventional model. SPSN, solitary pulmonary solid nodule

The diagnostic performance of three models in differentiating between benign and malignant SPSNs was shown in Table 2. ROC curves demonstrated that radiomics model (AUC_training_ = 0.96, AUC_testing_ = 0.96) outperformed conventional model (AUC_training_ = 0.88, AUC_testing_ = 0.86) (DeLong test, P_training_ < 0.01, P_testing_ < 0.05) in differentiating benign and malignant SPSNs in the training and testing datasets, while the integrated model was further slightly improved (AUC_training_ = 0.97, AUC_testing_ = 0.97) (Fig. 4 a, b), but there was no significant difference (DeLong test, P_training_ = 0.51, P_testing_ = 0.72) of discriminating ability between radiomics model and integrated model. DeLong test revealed that there was no statistical difference in the diagnostic efficacy of three models between the training and testing datasets with *p* values of 0.65, 0.84, and 0.89, respectively. Through the Hosmer-Lemeshow test, calibration curves indicated that there was a good fitness between prediction and observation for the discrimination probability of SPSNs (Fig. 4 c, d). Decision curves suggested that radiomics model and integrated model had higher clinical net benefit than conventional model (Fig. 4 e, f).

**Table 2:** Comparison of AUCs between the radiomics model, conventional model, and integrated model

Model	Cut-off	**Training dataset**				**Testing dataset**			
		AUC (95%CI)	SEN	SPE	ACC	AUC (95%CI)	SEN	SPE	ACC
Radiomics model	0.91	0.96 (0.925–0.996)	0.94	0.91	0.92	0.96 (0.914–0.996)	0.87	0.90	0.87
Conventional model	0.60	0.88 (0.823–0.941)	0.86	0.81	0.84	0.86 (0.767–0.948)	0.82	0.70	0.76
Integrated model	1.01	0.97 (0.940–0.997)	0.91	0.93	0.91	0.97 (0.928–1.000)	0.91	0.95	0.92

*AUC* Area under ROC curve, *95% CI* 95% confidence interval, *SEN* Sensitivity, *SPE* Specificity, *ACC* Accuracy, *Radiomics model* The model combining optimal radiomics scores based on 65 keV images of AP and VP, *Conventional model* The model based on significant clinical characteristics and spectral quantitative parameters, *Integrated model* The model combining radiomics model and Z_eff-AP_

## Discussion

The results of our study showed that radiomics scores based on 65 keV images of AP and VP from SDCT had the optimal performance within the range of 40-65 keV in the discrimination of benign and malignant SPSNs. Furthermore, we developed and validated a radiomics-based model based on optimal radiomics-based scores derived from 65 keV images of AP and VP, which had better diagnostic performance than conventional model, and the integrated model combining radiomics model and Z_eff-AP_ that was retained by multiple features screenings of clinical features and spectral quantitative parameters could further improve the discriminating ability slightly.

To the best of our knowledge, this is the first time to compare the performance of radiomics scores based on different keV VMIs of SDCT to distinguish benign and malignant SPSNs. Currently, in view of clinical need for image quality and resolution, we could obtain 161 monochromatic images between 40-200 keV from spectral CT. Low keV images could improve the density resolution of images and help optimize the display of low-contrast structures, as one of the important clinical applications of VMIs. Given that the reconstruction of 70 keV VMIs is roughly equivalent to a standard spectral CT acquisition performed at 120 kVp [31, 32], our study selected 40-65 keV VMIs to perform radiomics analysis. According to our results, radiomics-based scores varied with different energy levels, the higher energy level, the better diagnostic performance. We did not observe conceivable benefits of greater iodine-related attenuation at lower energy levels when noise tends to be constant low [17, 18] in our radiomics study. It was inconsistent with the assessment by Wen et al. [20] who confirmed that VMIs at 40 keV from SDCT may be an effective way to characterize solitary pulmonary nodules. It may be that overhigh contrast within the lesions covers subtle changes of features, which leads to the error of pixel information extraction in our radiomics study.

An additional important observation was that radiomics model was significantly better than conventional model, indicating the advantages of radiomics features obtained from SDCT in the identification of SPSNs. The top two radiomics features with the greatest relative weights obtained from training dataset in radiomics model were GLCM-Cluster-Prominence of AP and GLRLM-Short-Run-High-Gray-Level-Emphasis of VP. The former revealed larger asymmetry [33] and the latter revealed higher and more heterogeneous iodine uptake, and greater images roughness [34, 35], which indicated more complex histological architecture with malignant SPSNs. In our study, radiomics model also displayed higher diagnostic efficacy than previous conventional CT radiomics-based models regarding the qualitative diagnosis of pulmonary nodule [4, 5, 7–9], whose comparison was detailed in Supplementary Table A4. Among them, the deep learning model [4, 7], the machine learning model built with intranodular and perinodular features combined [4], and the contrast-enhanced CT radiomics-based model [5] all failed to increase more valid discriminating capability of the nature of SPSNs then spectral CT radiomics-based model. The above results may be attributed to two major points: Firstly, spatial and temporal alignment completely is ideal for data collection. Secondly, the combination of radiomics features in AP and VP can more comprehensively reflect the mixed distribution state of different nodules owing to vascular permeability or inflammatory components. It may be possible to contribute to selecting radiomics features, and increase the efficiency of identification in benign and malignant SPSNs. However, Zhuo et al. [6] generated a more predictive radiomics model in differentiating nature of SPSNs including adenocarcinoma and tuberculosis, which may be attributed to the fact that our study involved more clinically relevant samples with multiple types of SPSNs, not limited to the distinction between tuberculosis and adenocarcinoma. Besides, the patient population ratio of adenocarcinoma was lower than tuberculosis in their study, which went against the usual constituent ratio in clinical, in which pulmonary nodules with fewer signs of malignant are generally less likely to undergo a biopsy or surgery.

Moreover, we developed an integrated model based on radiomics model and Z_eff-AP_, which were combined together for the first time, the accuracy was improved from 87% to 92%, and the sensitivity and specificity were improved from 87% and 90% to 91% and 95%, respectively. Z_eff_ is a quantitative index derived from atomic number, representing the composite atom for a mixture or compound of various materials, and it can be calculated from dual-energy spectral computed tomography data [36] and applied to identify substance composition. The role of Z_eff_ in differentiating benign and malignant lung tumors was firstly reported by Gonzalez-perez, V. et al. [36]. Subsequently, the values of Z_eff_ in detecting tumor progression [37], evaluating histological types of lung cancer [21, 38], as well as taking part in gene expression [39] were discovered. The above results also remained that traditional spectral quantitative parameters may still reveal utility in differentiating benign and malignant SPSNs. Consequently, multi-dimensional consideration and analysis are required.

This study still has some limitations. Firstly, the sample size of our study was relatively small along with a large proportion of malignant SPSNs (75%) in our cohort, which may result in selection bias and exaggerate the diagnostic efficacy of predictive models to some extent, the efficacy of our models needs to be further validated in a large population. Secondly, the clinical application of our predictive models to general populations was limited to a single-center study, thus there is still a requirement for further verifying our models in a multi-center and independent validation cohort. Thirdly, no subgroup analysis of SPSNs was conducted in this study, further research based on spectral CT radiomics in the differentiation of tumor subtyping will be carried out. Finally, retrospective data collection may also lead to sample bias, and further prospective studies are still required.

## Conclusion

Among the 40-65 keV radiomics scores based on SDCT, 65 keV radiomics-based score had the optimal performance in distinguishing benign from malignant pulmonary nodules. The developed integrated model based on radiomics model and Z_eff-AP_ was significantly superior to conventional model in the discrimination of SPSNs. This method had the potential to reveal the heterogeneity of nodules and provided accurate information for the nature of SPSNs, which would serve to provide individual medical services for patients with SPSNs efficiently and scientifically.

## Supplementary Information

**Additional file 1:** **Table A1.** Pathologic results of the study population. **Table A2.** Comparison of diagnostic efficacy of radiomics scores based on 40-65keV images of arterial and venous phases and conventional 120kVp images from SDCT in the training and testing datasets. **Table A3.** Optimal radiomics features based on 65keV images of arterial and venous phases of SDCT. **Table A4.** General characteristics and main results of included studies in the discussion. **Figure A1.** Feature selection of LASSO regression in 65keV images of arterial phase. **Figure A2.** Feature selection of LASSO regression in 65keV images of venous phase. **Figure A3.** Heatmaps of the significant radiomics features derived from 65keV images. **Figure A4.** Bar chart for the radiomics model in the training (4a) and testing datasets (4b). **Figure A5.** Bar chart for the conventional model in the training (5a) and testing datasets (5b). **Figure A6.** Bar chart for the integrated model in the training (6a) and testing datasets (6b).

## Data Availability

Our study’s source code was uploaded at https://github.com/lszxuhang/zhuna-final.git. And the datasets generated during this study were available in the [Data of 242 patients] repository, [https://doi.org/10.6084/m9.figshare.21800861].
