# Supplementary material for: Spectral CT-based radiomics signature for distinguishing malignant pulmonary nodules from benign
Source: BMC Cancer. 2023 Jan 26;23:91. doi: 10.1186/s12885-023-10572-4 (PMC9878920; doi:10.1186/s12885-023-10572-4)
Supplement: Supplementary file 1 — Additional file 1: Table A1. Pathologic results of the study population. Table A2. Comparison of diagnostic efficacy of radiomics scores based on 40-65keV images of arterial and venous phases and conventional 120kVp images from SDCT in the training and testing datasets. Table A3. Optimal radiomics features based on 65keV images of arterial and venous phases of SDCT. Table A4. General characteristics and main results of included studies in the discussion. Figure A1. Feature selection of LASSO regression in 65keV images of arterial phase. Figure A2. Feature selection of LASSO regression in 65keV images of venous phase. Figure A3. Heatmaps of the significant radiomics features derived from 65keV images. Figure A4. Bar chart for the radiomics model in the training (4a) and testing datasets (4b). Figure A5. Bar chart for the conventional model in the training (5a) and testing datasets (5b). Figure A6. Bar chart for the integrated model in the training (6a) and testing datasets (6b). [file 12885_2023_10572_MOESM1_ESM.docx]

**Supplementary Materials**

**Study population:**

The study population was retrospectively enrolled solitary pulmonary solid nodules (SPSNs) patients who underwent contrast-enhanced dual-layer Spectral Detector CT (SDCT) examination within one month before surgery from our hospital between January 2016 and December 2020. Ultimately, 242 patients were included, whose pathological results were shown in **Table A1**.

**Radiological evaluation of SPSNs:**

In our study, the results of chest CT imaging indicating SPSNs were independently evaluated by two radiologists with more than 10 years of experience in chest imaging diagnosis using a picture archiving and communication system (PACS). A consensus was reached after mutual discussion in case of disagreements.

**Feature selection and optimal VMI sequence screening:**

We selected radiomics features with ICC >0.75 for subsequent analysis, after that a total of 107 stable features remained on 40-65keV images of arterial phase (AP), venous phase (VP), and conventional 120kVp, respectively. To improve the performance of the radiomics-based scores, a 10-fold cross validation of least absolute shrinkage and selection operator (LASSO) regression was carried out. A total of 14 radiomics-based scores were calculated respectively based on optimal radiomics features. The diagnostic performance of 14 radiomics-based scores was detailed in **Table A2**. Among these, the diagnostic performance based on 65 keV images in both AP and VP was the best in differentiating benign and malignant SPSNs in training and testing datasets, respectively. The LASSO algorithms of 65keV images of AP and VP were shown in **Figure A1** and **A2**, respectively. Heatmaps of the selected radiomics features derived from 65keV images were shown in **Figure A3**. The selected radiomics features of 65 keV radiomics-based scores in AP and VP were displayed in **Table A3**.

**Models building:**

In the process of building predictive models, significant clinical features and spectral CT quantitative parameters were selected to construct conventional model. The 14 radiomics-based scores were calculated respectively based on optimal radiomics features obtained from 40-65keV images of AP, VP, and conventional 120kVp; next, the optimal radiomics-based score was selected to establish radiomics model. Finally, an integrated model was built incorporating radiomics model with conventional model. Bar charts for radiomics model, conventional model, and integrated model were shown in **Figure A4, A5, and A6**.

**Comparison of radiomics models:**

In our study, spectral CT radiomics-based model displayed higher diagnostic efficacy than previous conventional CT radiomics-based models regarding the qualitative diagnosis of pulmonary nodules. Comparison of spectral CT radiomics-based model and conventional CT radiomics-based models mentioned in the part of discussion was detailed in **Table A4**.

**The calculation formula of three predictive models:**

The detailed calculation formula of conventional model, radiomics model, and integrated model was as follows:

**Score of conventional model** = 2.26+0.96×CEA-0.29×Z_eff-AP_+0.33×Years+1.27×CYFRA21-1+1.08×NSE+0.32×CT_40keV-VP_-0.80×NIC_AP_+1.75×NIC_VP_

**Radiomics score based on 65keV images of arterial phase = 1.85+0.29× shape_Flatness-0.50×shape_Elongation+3.61×shape_Maximum2DDiameterSlice-3.15×shape_Maximum2DDiameterRow-2.26×shape_SurfaceVolumeRatio-0.25×ngtdm_Busyness+1.82×gldm_LargeDependenceHighGrayLevelEmphasis+0.99×gldm_DependenceVariance+2.95×gldm_DependenceNonUniformityNormalized+1.85×glcm_Correlation-4.32×glcm_ClusterProminence-0.26×glcm_Autocorrelation-1.12×glcm_JointEnergy+1.87×glcm_Imc1-0.57×glszm_SizeZoneNonUniformity+1.20×firstorder_90Percentile+0.11×firstorder_Skewness+1.66×firstorder_InterquartileRange;**

**Radiomics score based on 65keV images of venous phase = 1.92-1.88×shape_SurfaceVolumeRatio+2.25×shape_Maximum2DDiameterSlice+0.49×shape_Flatness+0.10×ngtdm_Strength+0.72×gldm_DependenceNonUniformityNormalized-0.81×glcm_ClusterShade-1.55×glcm_InverseVariance-1.20×glcm_DifferenceVariance+0.92×glcm_Imc1-1.22×glrlm_RunVariance+0.59×glrlm_RunEntropy-2.53×glrlm_ShortRunHighGrayLevelEmphasis-1.10×glszm_LargeAreaHighGrayLevelEmphasis+1.59×glszm_HighGrayLevelZoneEmphasis-0.82×glszm_SmallAreaLowGrayLevelEmphasis-0.96×firstorder_Energy-0.55×firstorder_Range+0.48×firstorder_90Percentile;**

**Score of radiomics model** = -0.34+0.65×radiomics score based on 65keV images of arterial phase +0.56×radiomics score based on 65keV images of venous phase.

**Score of integrated model** =-22.75+1.17×Score of radiomics model +2.86×Z_eff-AP_.

**Table A1. Pathologic results of the study population**

| Pathology | | Frequency^†^ |
| --- | --- | --- |
| Benign SPSNs | Inflammatory granuloma | 9.9 (24/242) |
|  | Tuberculosis | 7.9 (19/242) |
|  | Hamartoma | 4.5 (11/242) |
|  | inflammatory pseudotumor | 1.2 (3/242) |
|  | pulmonary sclerosing pneumocytoma | 0.8 (2/242) |
|  | Others | 0.8 (2/242) |
| Malignant SPSNs | Adenocarcinoma | 57.4 (139/242) |
|  | small cell lung cancer | 9.1 (22/242) |
|  | Squamous cell carcinoma | 8.3 (20/242) |

Note: ^†^Data expressed as percentage, with numerator and denominator in parentheses. SPSN, solitary pulmonary solid nodule

**Table A2. Comparison of diagnostic efficacy of radiomics scores based on 40-65keV images of arterial and venous phases and conventional 120kVp images from SDCT in the training and testing datasets**

| Scores | training dataset | | | | |  | testing dataset | | | |
| --- | --- | --- | --- | --- | --- | --- | --- | --- | --- | --- |
|  | AUC (95% CI) | ACC | | SEN | SPE |  | AUC (95% CI) | ACC | SEN | SPE |
| A40 | 0.74(0.664-0.810) | | 0.72 | 0.75 | 0.64 |  | 0.69(0.566-0.803) | 0.62 | 0.60 | 0.68 |
| A45 | 0.82(0.756, 0.876) | | 0.82 | 0.90 | 0.60 |  | 0.79(0.683, 0.892) | 0.81 | 0.91 | 0.53 |
| A50 | 0.85(0.783, 0.904) | | 0.78 | 0.78 | 0.79 |  | 0.81(0.707, 0.894) | 0.73 | 0.73 | 0.74 |
| A55 | 0.88(0.827, 0.930) | | 0.82 | 0.83 | 0.79 |  | 0.85(0.744, 0.934) | 0.80 | 0.85 | 0.63 |
| A60 | 0.92(0.884, 0.956) | | 0.88 | 0.89 | 0.86 |  | 0.85(0.755, 0.937) | 0.81 | 0.85 | 0.68 |
| A65 | 0.94(0.902, 0.971) | | 0.89 | 0.90 | 0.88 |  | 0.92(0.843, 0.979) | 0.88 | 0.87 | 0.89 |
| Ac | 0.90(0.843, 0.943) | | 0.85 | 0.85 | 0.83 |  | 0.86(0.769, 0.929) | 0.78 | 0.78 | 0.79 |
| V40 | 0.81(0.749, 0.869) | | 0.77 | 0.80 | 0.67 |  | 0.74(0.629, 0.851) | 0.73 | 0.80 | 0.53 |
| V45 | 0.89(0.844, 0.933) | | 0.73 | 0.67 | 0.93 |  | 0.84(0.749, 0.920) | 0.69 | 0.62 | 0.89 |
| V50 | 0.91(0.859, 0.944) | | 0.79 | 0.75 | 0.90 |  | 0.88(0.772, 0.960) | 0.76 | 0.69 | 0.95 |
| V55 | 0.89(0.844, 0.937) | | 0.86 | 0.89 | 0.76 |  | 0.87(0.773, 0.949) | 0.86 | 0.95 | 0.63 |
| V60 | 0.91(0.866, 0.952) | | 0.85 | 0.85 | 0.83 |  | 0.86(0.775, 0.931) | 0.82 | 0.84 | 0.79 |
| V65 | 0.92(0.867, 0.967) | | 0.93 | 0.98 | 0.81 |  | 0.88(0.793, 0.954) | 0.84 | 0.87 | 0.74 |
| Vc | 0.76(0.681, 0.834) | | 0.81 | 0.87 | 0.62 |  | 0.64(0.499, 0.771) | 0.77 | 0.91 | 0.37 |

Note: A40-65, radiomics scores based on 40-65keV images at 5 keV intervals of arterial phase; V40-65, radiomics scores based on 40-65keV images at 5 keV intervals of venous phase; Ac, radiomics scores based on conventional 120kVp images of arterial phase; Vc, radiomics scores based on conventional 120kVp images of venous phase; SDCT, dual-layer Spectral Detector CT; ACC, accuracy; SEN, sensitivity; SPE, specificity

**Table A3. Optimal radiomics features based on 65keV images of arterial and venous phases of SDCT**

| Radiomics features | 65keV-AP | 65keV-VP |
| --- | --- | --- |
| Shape          NGTDM  GLDM      GLCM  GLSZM  GLRLM  Firstorder | shape_Flatness  shape_Elongation  shape_Maximum2DDiameterSlice  shape_Maximum2DDiameterRow  shape_SurfaceVolumeRatio  ngtdm_Busyness  gldm_LargeDependenceHighGrayLevelEmphasis  gldm_DependenceVariance  gldm_DependenceNonUniformityNormalized  glcm_Correlation  glcm_ClusterProminenc  glcm_Autocorrelation  glcm_JointEnergy  glcm_Imc1  glszm_SizeZoneNonUniformity  firstorder_90Percentile  firstorder_Skewness  firstorder_InterquartileRange | shape_SurfaceVolumeRatio  shape_Maximum2DDiameterSlice  shape_Flatness  ngtdm_Strength  gldm_DependenceNonUniformityNormalized  glcm_ClusterShade  glcm_InverseVariance  glcm_DifferenceVariance  glcm_Imc1  glszm_LargeAreaHighGrayLevelEmphasis  glszm_HighGrayLevelZoneEmphasis  glszm_SmallAreaLowGrayLevelEmphasis  glrlm_RunVariance  glrlm_RunEntropy  glrlm_ShortRunHighGrayLevelEmphasis  firstorder_Energy  firstorder_Range  firstorder_90Percentile |

Note: NGTDM, neighborhood gray tone difference matrix; GLDM, gray-level dependence matrix; GLCM, gray-level co-occurrence matrix; GLSZM, gray-level size zone matrix; GLRLM, Gray Level Run Length Matrix; AP, arterial phase; VP, venous phase

**Table A4: General characteristics and main results of included studies in the discussion.**

| **Study ID** | **No. nodules** | **Type of nodules** | **CT Scanner** | **Tube voltage/ Tube current** | **Slice thickness** | **Main Results** |
| --- | --- | --- | --- | --- | --- | --- |
| Beig, N.  2019 [1] | 290 | Solitary pulmonary nodules | Siemens (Sygno; Erlangen, Germany), General Electric (Lightspeed16; Waukesha, Wis), Philips (iCT; Cleveland, Ohio), Toshiba (Aquilion; Tochigi-ken, Japan) | 120-140 kV/- | 1.0–6.5 mm | Perinodular Classifier: AUC=0.75; Sen=0.77; Spe=0.63; Acc=0.68  Intranodular Classifier: AUC=0.75; Sen=0.75; Spe=0.61; Acc=0.65  Combined Classifier: AUC=0.80; Sen=0.74; Spe=0.68; Acc=0.71  Deep learning model: AUC=0.76; Sen=0.69; Spe=0.71; Acc=0.70 |
| Yang, X.  2018 [2] | 302 | SPSN | Definition AS+ 128-Slice; Siemens Healthcare, Germany | 120 kV/ automatic tube current modulation | 2mm | PR (Plain radiomics model): AUC=0.776; Sen=0.778; Spe=0.643  VR (Vein radiomics model): AUC=0.769; Sen=0.810; Spe=0.643  PVR (Plain radiomics & Vein radiomics model): AUC=0.798; Sen=0.810; Spe=0.571  PRC (Plain radiomics & Clinical factor model): AUC=0.815; Sen=0.825; Spe=0.643  VRC (Vein radiomics & Clinical factor model): AUC=0.841; Sen=0.825; Spe=0.75  PVRC (Plain radiomics & Vein radiomics & Clinical factors model): AUC=0.837; Sen=0.825; Spe**=**0.607 |
| Zhuo, Y.  2021 [3] | 313 | SPSN | Somaton Force (SIEMENS, Germany), Aquilion One/320 (TOSHIBA, Japan) | 120 kV/ auto mA | ＜2mm | Clinical model: AUC=0.91; Sen=0.9245; Spe=0.6000; Acc=0.7849  Radiomics signature: AUC=0.99; Sen=0.9643; Spe=0.9385; Acc=0.9462  Radiomics nomogram: AUC=0.99; Sen**=**0.9841; Spe**=**0.9000; Acc=0.9570 |
| Feng, B.  2020 [4] | 550 | SPSN | Siemens (Sygno; Erlangen, Germany), General Electric (Lightspeed 16; Waukesha, Wis), Toshiba (Aquilion; Tochigi-ken, Japan) | 120 kV/ mAs settings were dependent on the machine used and adjusted accordingly | 1.0-3.0 mm | Clinical model: AUC=0.672; Sen=0.603; Spe=0.726; Acc=0.635  Deep learning signature: AUC=0.771; Sen=1; Spe=0.510; Acc=0.87  Deep learning nomogram: AUC=0.809; Sen**=**0.908; Spe**=**0.608; Acc=0.828 |
| Feng, B.  2020 [5] | 426 | SPSN | dual-energy Somatom Flash (Siemens Medical Systems, Forchheim, Germany), 64-detector-row CT scanner Aquilion One (Toshiba Medical Systems, Otawara, Japan) | 120 kV/ auto mA | 1.0-3.0 mm | Clinical model: AUC=0.5961; Sen=0.4514; Spe=0.7632; Acc=0.5165  Radiomics signature: AUC=0.8735; Sen=0.8681; Spe=0.7632; Acc=0.8462  Radiomics nomogram: AUC=0.9064; Sen**=**0.7917; Spe**=**0.9474; Acc=0.8242 |
| Liu, A.  2020 [6] | 875 | benign and malignant pulmonary nodules | SIEMENS SOMATOM Definition Flash system (Siemens Healthineers, Erlangen, Germany) | 120 kV/ 150 effective mAs | 1.0 mm | Clinical variables model: AUC=0.663  Clinical variables + Radiomics score model: AUC=0.809 |

Note: All studies were retrospectively designed. Internal validation cohort results in single-center studies and external validation cohort results in multi-center studies were included in the table. SPSN, solitary pulmonary solid nodule; Sen, Sensitivity; Spe, Specificity; Acc, Accuracy; "-" indicates not mentioned


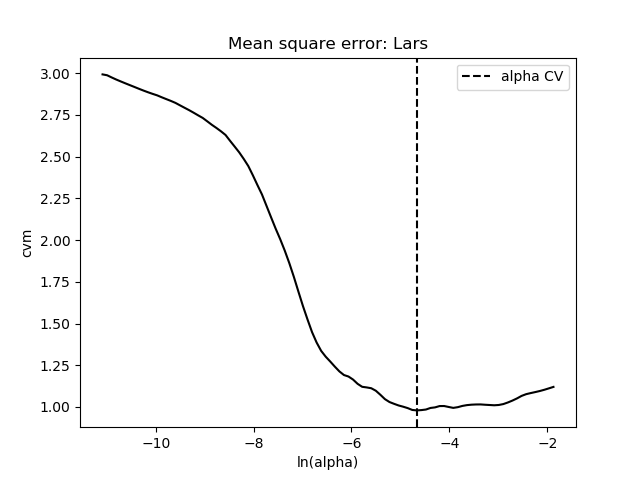

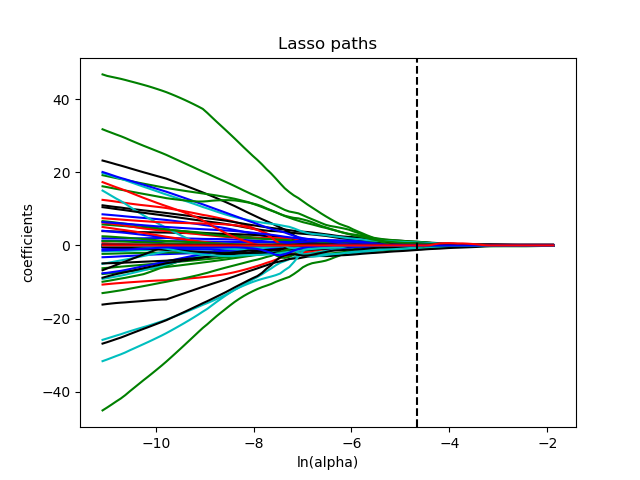


**1b**

**1a**

**Figure A1:** Feature selection of LASSO regression in 65keV images of arterial phase. The LASSO regression could reduce the feature dimension by shrinking the coefficients of some features to zero and further help to select significant features with non-zero coefficient. To improve the performance of the radiomics-based score in 65keV images of arterial phase, a 10-fold cross validation was carried out. The best penalty factor was obtained during the cross-validation procedure with the minimum cross-validation error as 0.0095, and the deviation was 0.51 (1a). After LASSO algorithm, a total of 18 features were remained (1b). LASSO, least absolute shrinkage and selection operator


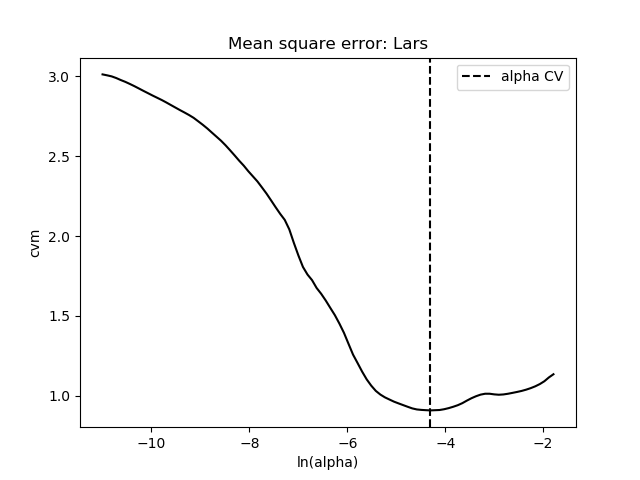

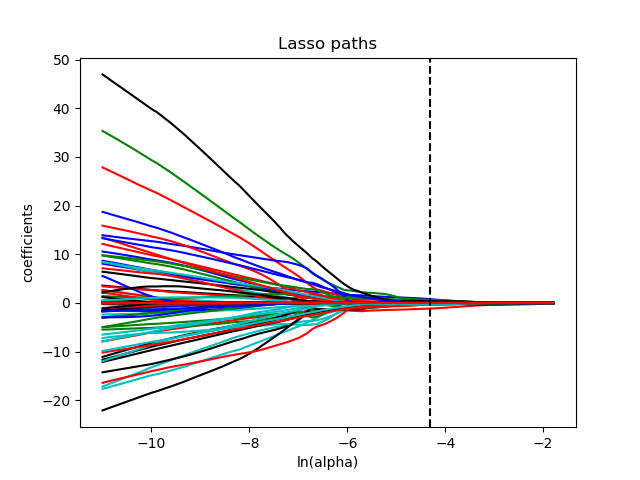


**2b**

**2a**

**Figure A2:** Feature selection of LASSO regression in 65keV images of venous phase. The LASSO regression could reduce the feature dimension by shrinking the coefficients of some features to zero and further help to select significant features with non-zero coefficient. To improve the performance of the radiomics-based score in 65keV images of venous phase, a 10-fold cross validation was carried out. The best penalty factor was obtained during the cross-validation procedure with the minimum cross-validation error as 0.0135, and the deviation was 0.89 (2a). After LASSO algorithm, a total of 18 features were remained (2b). LASSO, least absolute shrinkage and selection operator


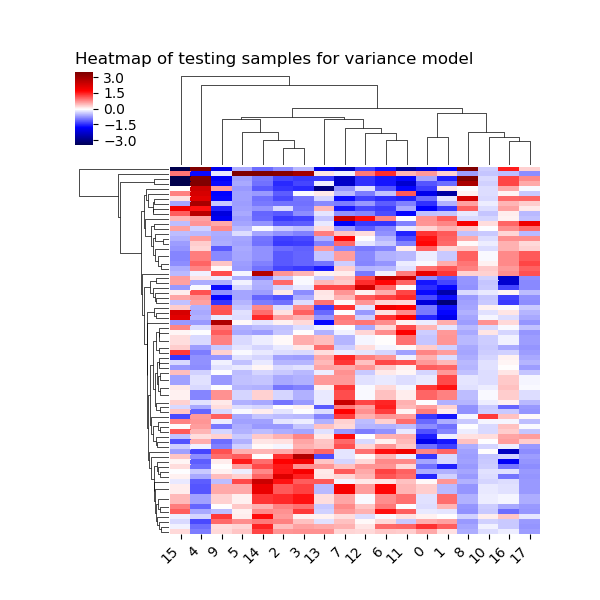

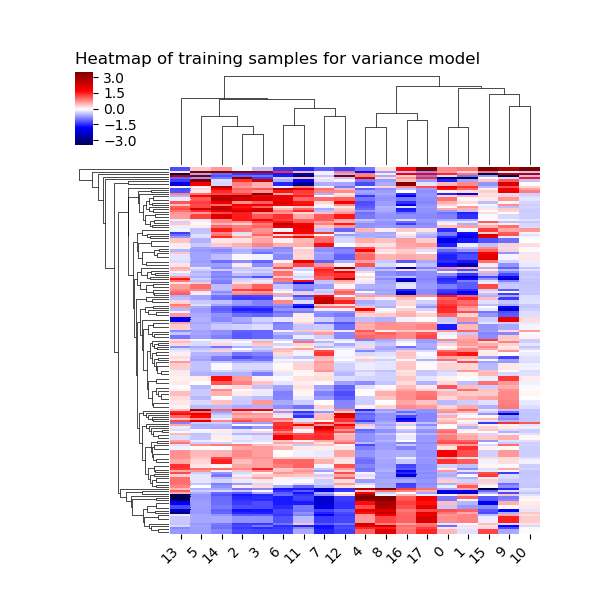

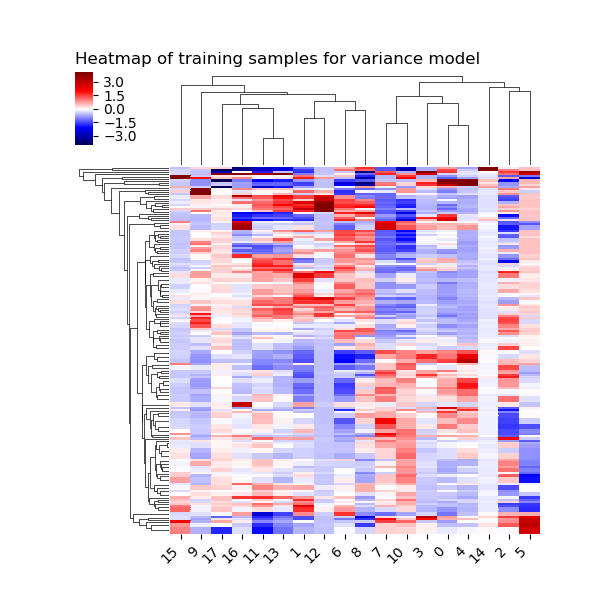

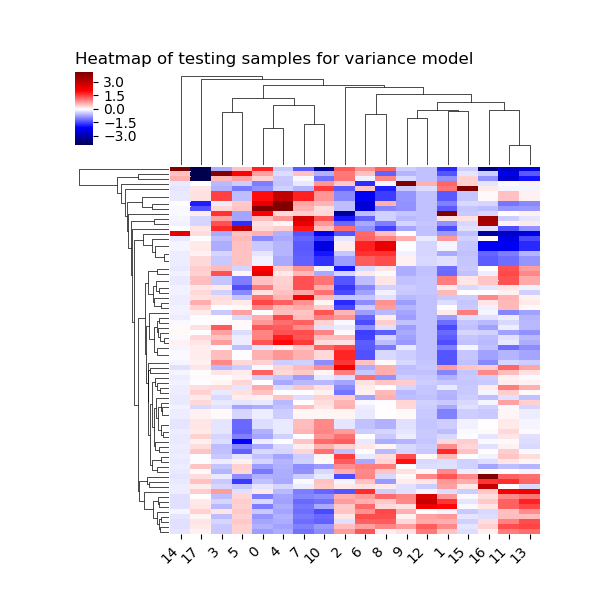


3a

3d

3b

3c

**Figure A3:** Heatmaps of the significant radiomics features derived from 65keV images. Heatmaps of 65keV radiomics-based score of arterial phase in training (3a) and testing datasets (3b). Heatmaps of 65keV radiomics-based score of venous phase in training (3c) and testing datasets (3d). Each row corresponds to one patient, and each column corresponds to the selected radiomics features


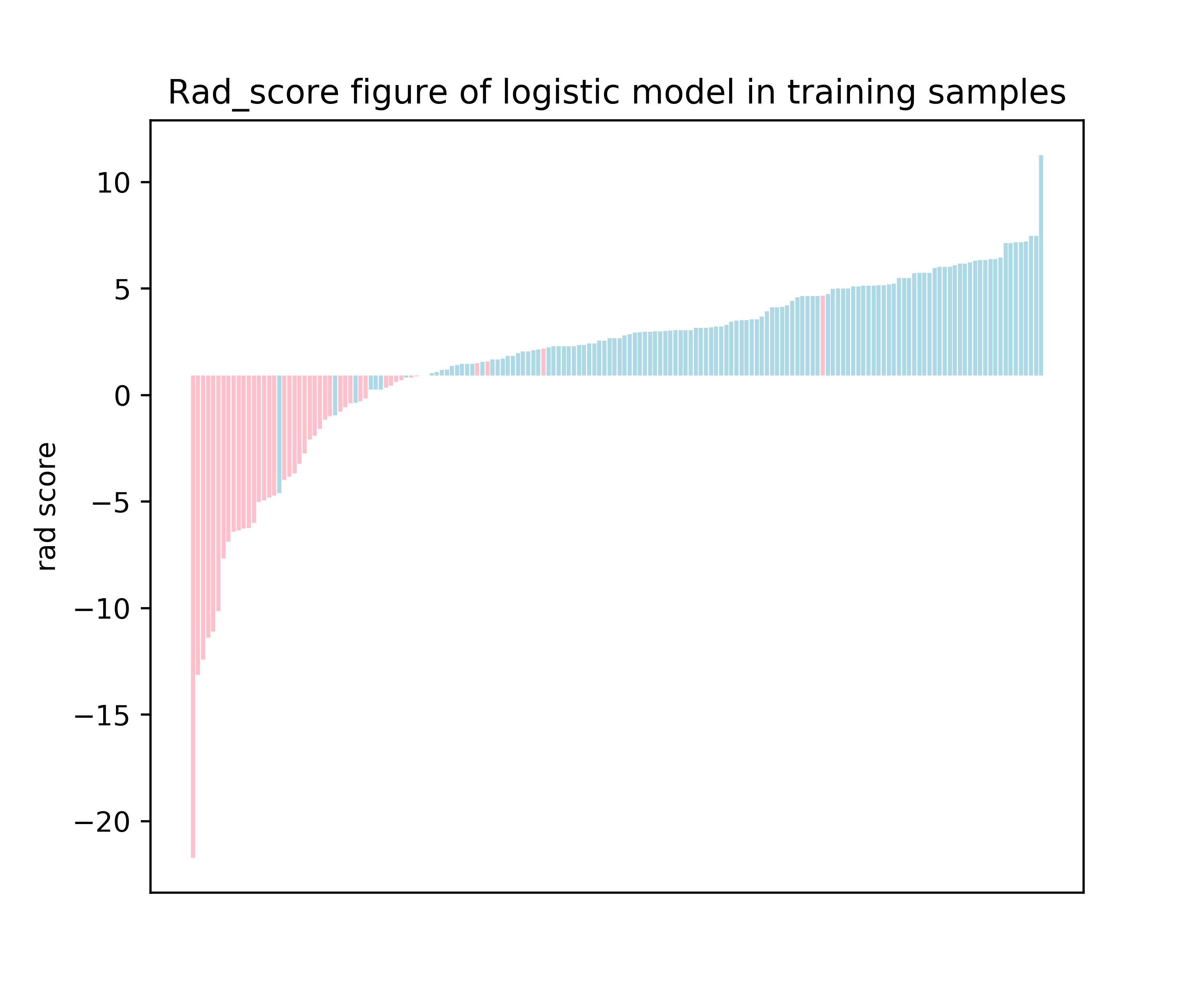

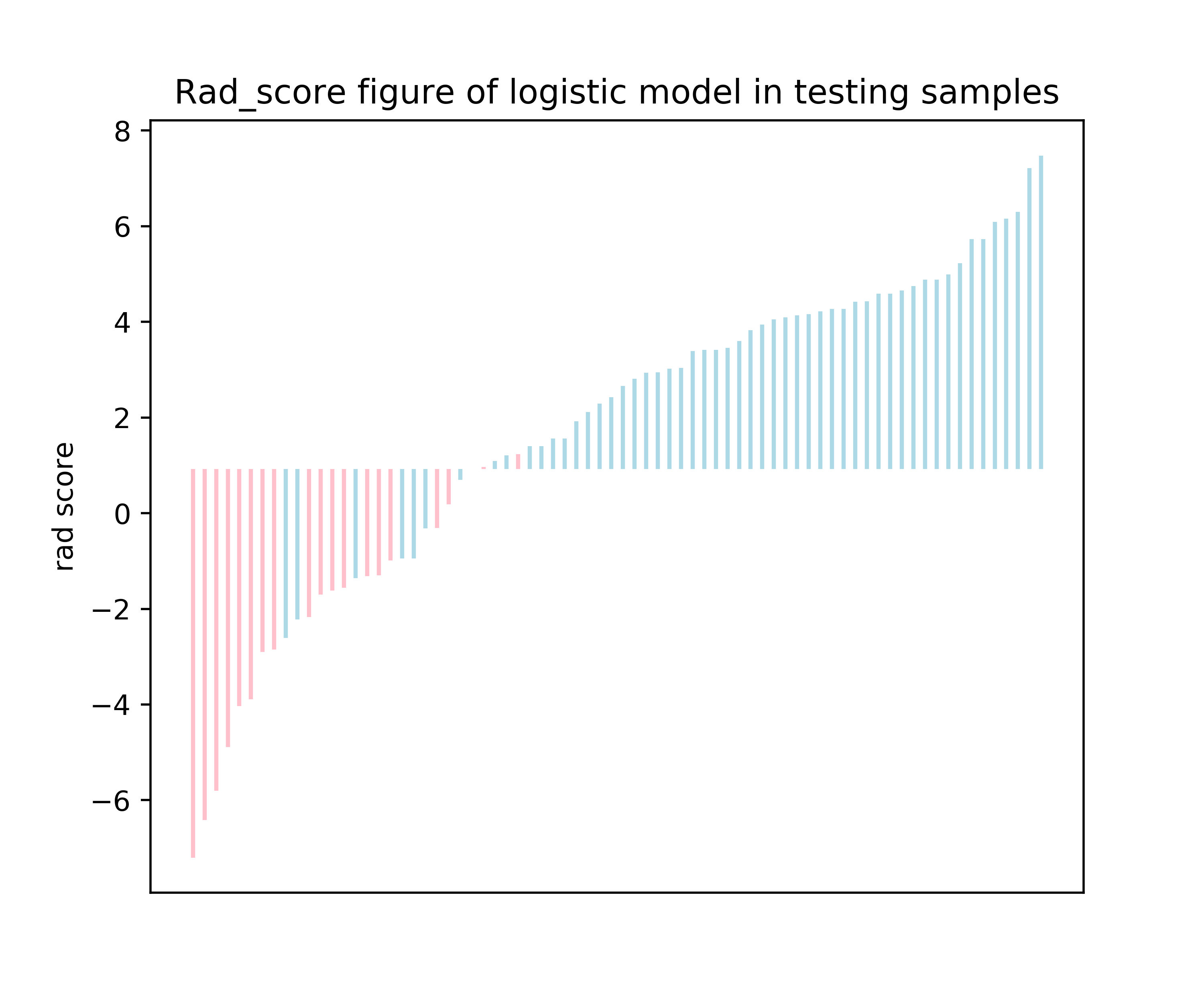


**4b**

**4a**

**Figure A4:** Bar chart for the radiomics model in the training (4a) and testing datasets (4b). Blue and pink bars refer to actual malignant and benign SPSNs, respectively. Up and down bars refer to the predicted malignant and benign SPSNs, respectively. Rad-score, the score of spectral CT-based radiomics model combining optimal radiomics scores based on 65keV images of arterial phase and venous phase


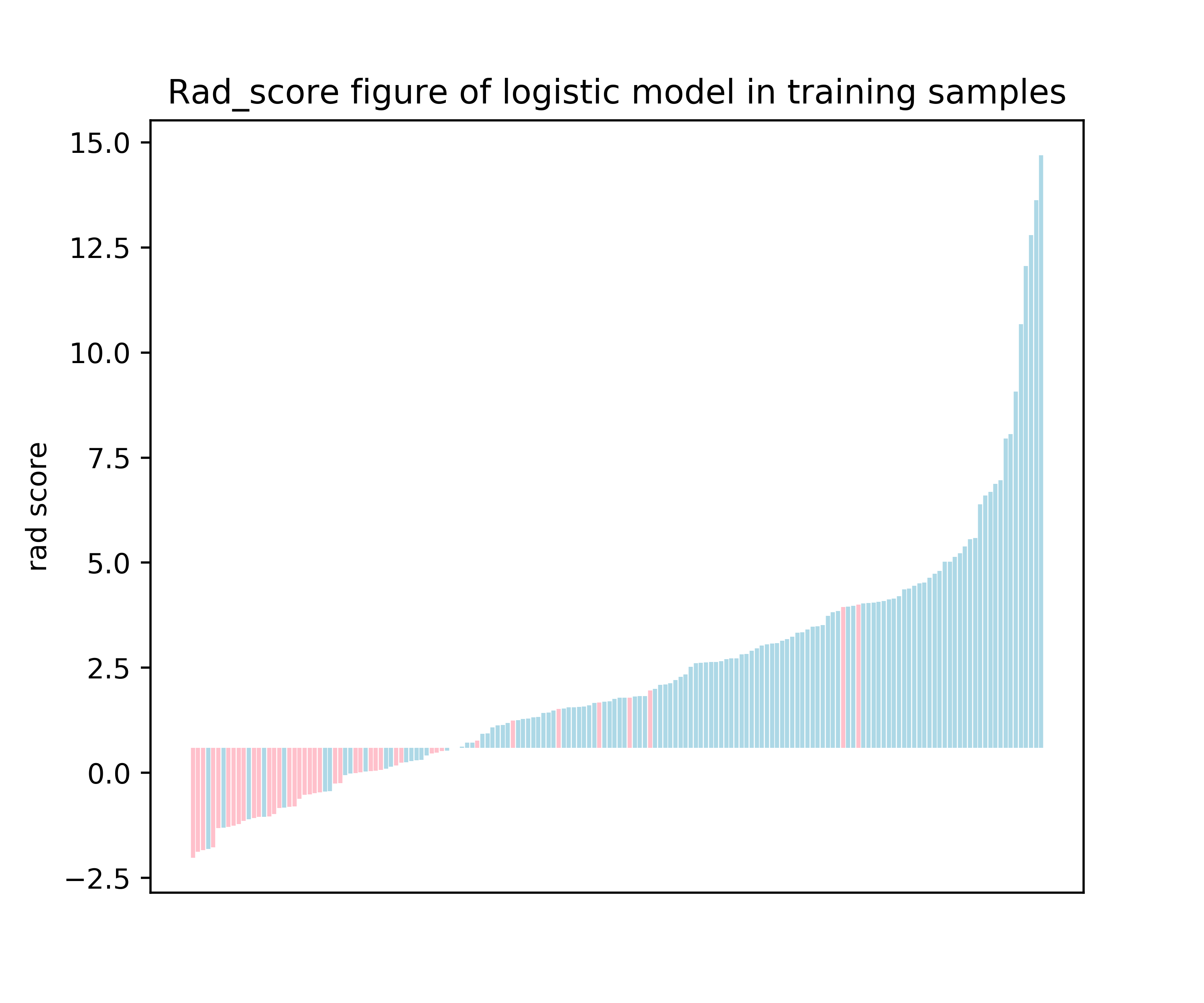

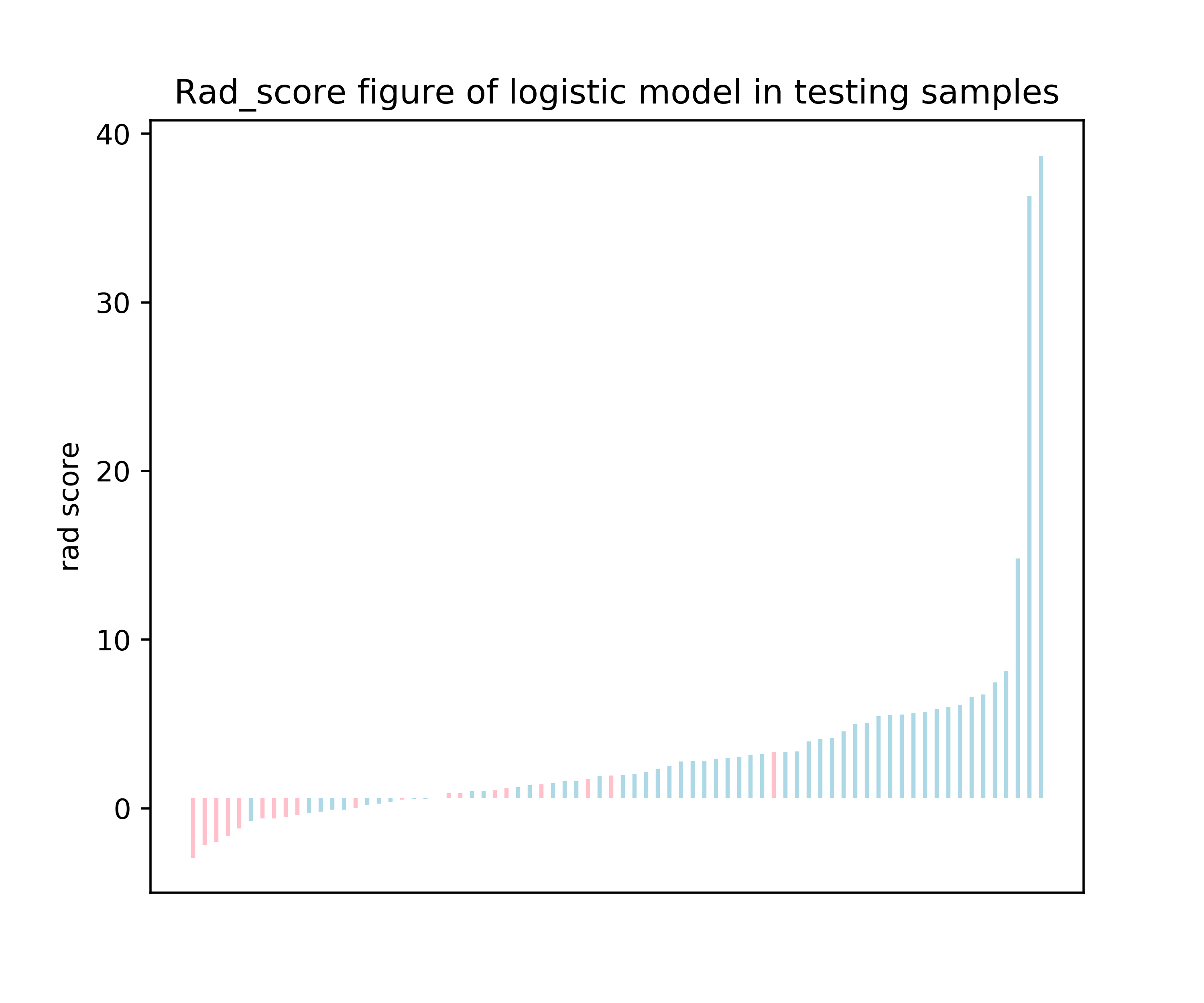


**5b**

**5a**

**Figure A5:** Bar chart for the conventional model in the training (5a) and testing datasets (5b). Blue and pink bars refer to actual malignant and benign SPSNs, respectively. Up and down bars refer to the predicted malignant and benign SPSNs, respectively. Rad-score, the score of conventional model based on significant clinical characteristics and spectral quantitative parameters


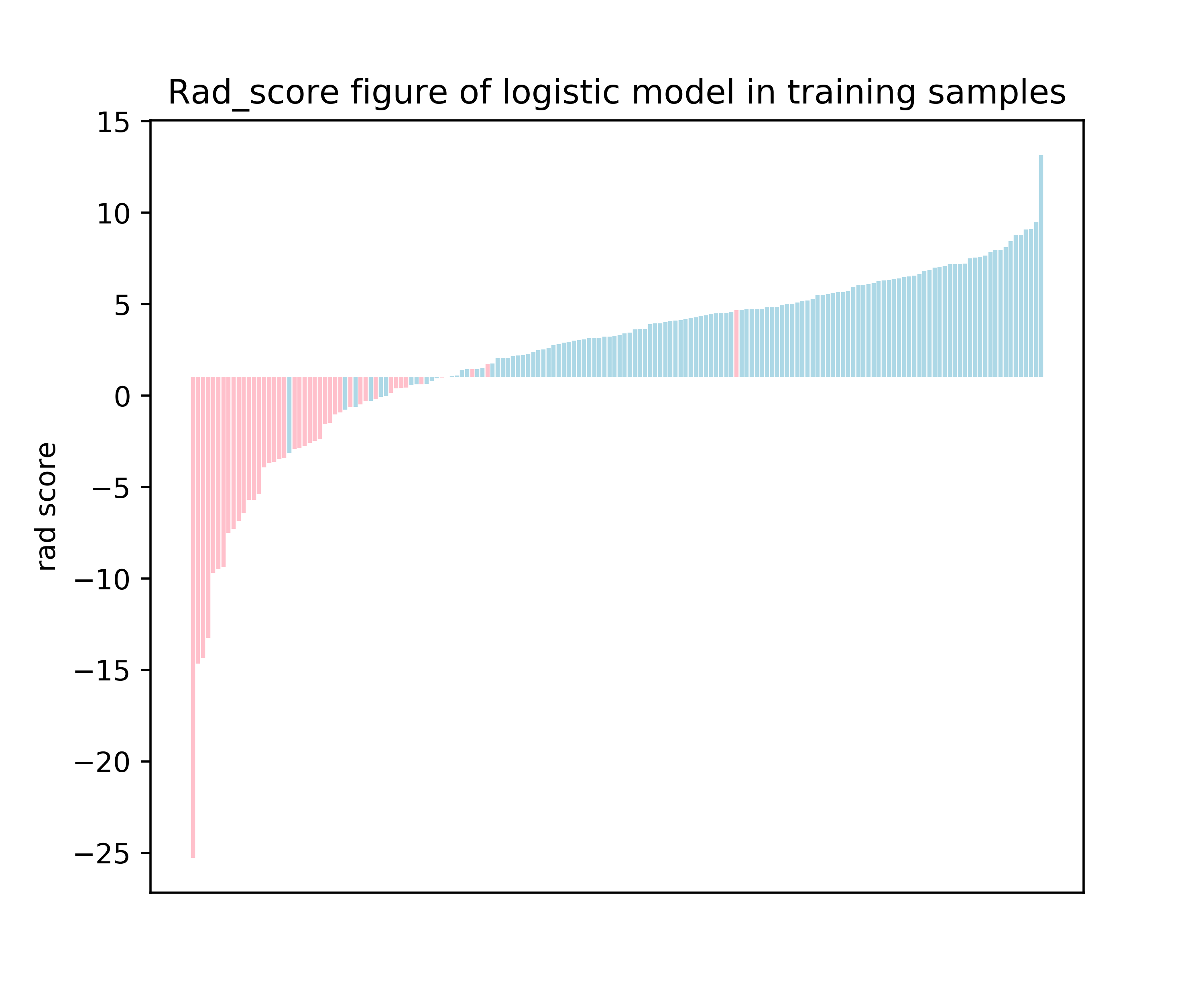

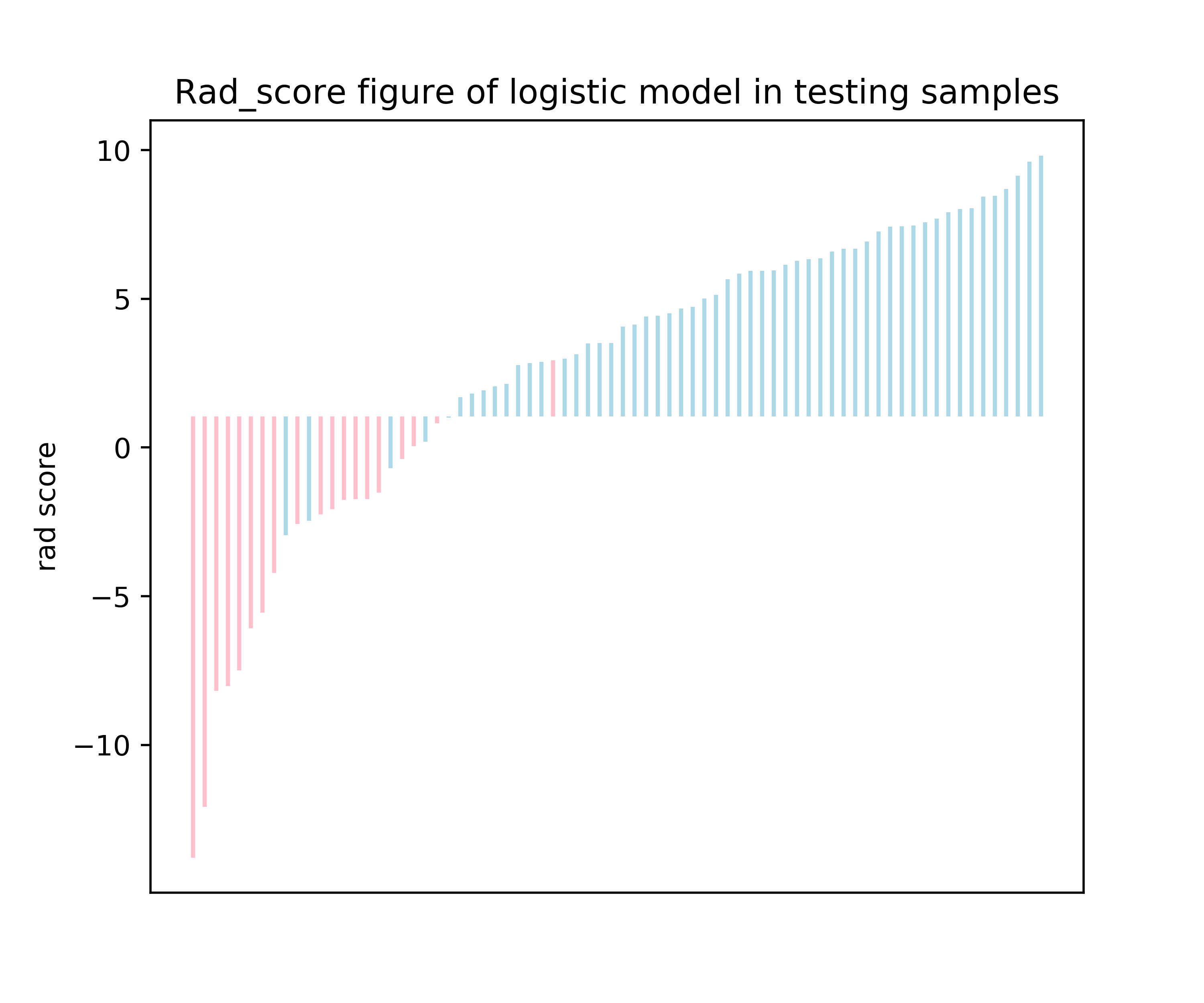


**6b**

**6a**

**Figure A6:** Bar chart for the integrated model in the training (6a) and testing datasets (6b). Blue and pink bars refer to actual malignant and benign SPSNs, respectively. Up and down bars refer to the predicted malignant and benign SPSNs, respectively. Rad-score, the score of integrated model combining radiomics model and Z_eff-AP_

**References:**

[1] N. Beig, M. Khorrami, M. Alilou, P. Prasanna, N. Braman, M. Orooji, S. Rakshit, K. Bera, P. Rajiah, J. Ginsberg, C. Donatelli, R. Thawani, M. Yang, F. Jacono, P. Tiwari, V. Velcheti, R. Gilkeson, P. Linden, A. Madabhushi, Perinodular and Intranodular Radiomic Features on Lung CT Images Distinguish Adenocarcinomas from Granulomas, Radiology 290(3) (2019) 783-792.https://doi.org/10.1148/radiol.2018180910.

[2] X. Yang, J. He, J. Wang, W. Li, C. Liu, D. Gao, Y. Guan, CT-based radiomics signature for differentiating solitary granulomatous nodules from solid lung adenocarcinoma, Lung Cancer 125 (2018) 109-114.https://doi.org/10.1016/j.lungcan.2018.09.013.

[3] Y. Zhuo, Y. Zhan, Z. Zhang, F. Shan, J. Shen, D. Wang, M. Yu, Clinical and CT Radiomics Nomogram for Preoperative Differentiation of Pulmonary Adenocarcinoma From Tuberculoma in Solitary Solid Nodule, Front Oncol 11 (2021) 701598.https://doi.org/10.3389/fonc.2021.701598.

[4] B. Feng, X. Chen, Y. Chen, S. Lu, K. Liu, K. Li, Z. Liu, Y. Hao, Z. Li, Z. Zhu, N. Yao, G. Liang, J. Zhang, W. Long, X. Liu, Solitary solid pulmonary nodules: a CT-based deep learning nomogram helps differentiate tuberculosis granulomas from lung adenocarcinomas, Eur Radiol 30(12) (2020) 6497-6507.https://doi.org/10.1007/s00330-020-07024-z.

[5] B. Feng, X. Chen, Y. Chen, K. Liu, K. Li, X. Liu, N. Yao, Z. Li, R. Li, C. Zhang, J. Ji, W. Long, Radiomics nomogram for preoperative differentiation of lung tuberculoma from adenocarcinoma in solitary pulmonary solid nodule, Eur J Radiol 128 (2020) 109022.https://doi.org/10.1016/j.ejrad.2020.109022.

[6] A. Liu, Z. Wang, Y. Yang, J. Wang, X. Dai, L. Wang, Y. Lu, F. Xue, Preoperative diagnosis of malignant pulmonary nodules in lung cancer screening with a radiomics nomogram, Cancer Commun (Lond) 40(1) (2020) 16-24. https://doi.org/10.1002/cac2.12002.
